# When to stop reviewing: validation of stop criteria in ASReview

**DOI:** 10.1186/s12874-026-02866-5

**Published:** 2026-05-09

**Authors:** C. Kempny, K. Annac, D. Wahidie, Y. Yilmaz-Aslan, P. Brzoska

**Affiliations:** Health Services Research, Witten/Herdecke University, Faculty of Health, School of Medicine, Witten, Germany

**Keywords:** Systematic reviews, Title and abstract screening, ASReview, Stop Criteria, Machine learning, Screening efficiency

## Abstract

**Background:**

Systematic reviews are essential for evidence-based research, but they often require a great deal of time and effort. Although title and abstract (T&A) screening is just one part of the review process, it can be very time-consuming when search strategies retrieve large numbers of records. Given the exponential growth of scientific publications in recent decades, tools such as ASReview, which use machine learning (ML) for active learning-based screening, aim to reduce the workload. However, since ASReview helps users prioritise potentially relevant studies rather than supporting them in screening all records, a key question arises: at what point can the screening process safely stop without risking the omission of relevant studies when the entire dataset is not being reviewed?

**Methods:**

This simulation study tested three proposed stop criteria for terminating screening in ASReview without loss of relevant data: (1) stopping after a calculated number of relevant studies based on an initial sample; (2) stopping after a fixed number of consecutively studies deemed irrelevant; (3) stopping after a predefined percentage of the dataset has been screened. A total of 35,000 automated title and abstract screenings were conducted using five datasets from the SYNERGY repository. Key outcomes included the percentage of studies screened until the last relevant study was found and the number of relevant studies missed under each stop criterion.

**Results:**

The proportion of the dataset that needed to be screened to identify all relevant studies (as pre-classified in the SYNERGY dataset) varied greatly across datasets, ranging from 2.9% to 76.9% on average. None of the tested stop criteria could consistently identify all relevant studies across all datasets. Stop criterion 1 was reliable in only 2% of simulations. Stop criterion 2 showed high variability, with thresholds ranging from 2% to 61%, depending on the dataset. Stop criterion 3 failed to define a universal percentage applicable across datasets.

**Conclusions:**

ASReview can reduce screening workload by prioritizing potentially relevant studies through ML-based ranking, thereby allowing researchers to identify relevant studies earlier in the screening process. However, no stop criterion reliably ensures that all relevant studies are identified. Early stopping may result in missed studies, depending on dataset characteristics. Current stop criteria should be applied cautiously and potentially combined with quality assurance measures. Further research is needed to develop more robust and generalizable stopping rules.

**Trial registration:**

Not applicable – this is a simulation study, not a registered systematic review.

**Supplementary Information:**

The online version contains supplementary material available at 10.1186/s12874-026-02866-5.

## Background

 Systematic Reviews play a key role in evidence-based research [1]. They form the basis for clinical guidelines, theoretical model development, and evidence-based policy decisions in a wide range of disciplines such as health research [2], as non-systematic approaches to a literature search may introduce bias and produce misleading conclusions [3]. Systematic reviews form the basis for new care concepts, treatment methods, drug development, as well as for the systematic evaluation of the effectiveness and efficiency of health interventions, following established review standards such as those proposed by the Cochrane Collaboration [4]. However, the screening process for systematic reviews is very laborious and time-consuming [5], requiring significant resources from researchers. Title and abstract screening (T&A screening) alone can take a long time if there are a large number of abstracts to be screened [5]. The number of records retrieved and thus the subsequent screening burden is heavily influenced by the specificity and precision of the underlying search strategy: broader search strategies increase recall but also generate larger volumes of records requiring manual review.

The number of published studies has increased considerably over time [6]. As a result, the number of studies found in search databases for reviews can become so high that manual screening becomes considerably more difficult [7]. Due to the large amount of time required to compile a methodically adequate review, the results collected are sometimes already outdated by the time of publication [7]. A more precise search strategy, ideally developed in collaboration with a specialized search librarian, can reduce the effort required in the screening process and thus ensure faster publication of the results. However, depending on how the search is refined, it may also lead to the unintentional exclusion of important publications before they even reach the screening process [8]. Using software, text mining and artificial intelligence (AI) in the review process can help to reduce the workload and prevent relevant studies from being overlooked [9]. Researchers are increasingly seeking out these forms of support [2]. Various tools are available to support literature search, duplicate detection, T&A screening, and the resolution of screening conflicts. By pre-sorting the results according to their potential relevance, some tools use text mining and AI to reduce the screening workload [7, 9]. Several platforms have been developed to facilitate this process including Rayyan or Covidence. While these tools assist in organizing the screening process by filtering studies, more advanced AI-based tools can improve efficiency by dynamically adjusting the selection of studies as the process progresses. One example of such a tool is ASReview [7, https://asreview.nl].

The screening process in ASReview [8] is based on an active learning algorithm (a machine learning (ML) approach in which the model is iteratively trained on user decisions) that helps users find relevant studies in a large data pool. In this context, AI refers to the broader concept of computational systems performing tasks that typically require human judgment, while ML specifically denotes the statistical algorithms that learn patterns from labeled data. In case of ASReview, first, a small set of studies is manually screened at the title and abstract level to train the model. Based on the features extracted from the titles and abstracts (e.g., term frequency–inverse document frequency vectors), ASReview then suggests studies for review based on previous screening decisions, showing the most promising entries first. The system uses ML to continuously learn from each screening decision and dynamically adjust the ranking of studies. As more studies are screened, the model becomes more accurate. The tool therefore re-ranks (sorts) the remaining unscreened dataset after each screening decision, so that studies predicted as most likely relevant appear at the top and those predicted as irrelevant appear at the bottom. The actual inclusion or exclusion decision remains with the human reviewer; the ML model only determines the order in which studies are presented. Various tests of ASReview have shown that potentially relevant studies tend to be displayed at the beginning of the screening and that few or no relevant studies appear further down the ranked data set [9, 10]. For this reason, it may be feasible to stop the screening process early, since most or all relevant studies may be identified at the beginning. However, this assumption may hold more strongly for reviews with well-defined, narrow research questions (e.g., simple PICO frameworks) where the relevant studies share similar characteristics; for broader or more complex review questions, the ML model may be less effective at clustering relevant studies early in the ranking. The stopping process itself and any automation of this process must be critically examined, as errors may occur in the ranking of studies by the ML model, potentially leading to relevant studies being presented later in the screening process or being missed if screening is stopped prematurely [7, 11].

There are several feasible options for an appropriate criterion to stop the screening process. For example, researchers could set a numerical threshold and stop the review process after a certain number of relevant studies, assuming that all relevant studies have already been proposed in the review process. One proposed method for ending T&A screening early is the SYMBALS Core methodology [12]. In addition to various process steps for quality assurance of the review process, two stop criteria are proposed in SYMBALS Core that could end a screening process early in ASReview. The first criterion is to stop the review when the estimated number of all potentially eligible studies has been reached. This number is estimated beforehand using a small sample of the studies and extrapolated to the total data set. In practice, this works as follows: suppose the full dataset contains 10,000 studies and a random sample of 100 is reviewed manually before ASReview is started. If five of those 100 studies are found to be relevant, the researcher can extrapolate that approximately 500 (5%) of the total 10,000 studies are relevant, at which point they stop screening. However, this estimation is inherently imprecise, as the true proportion of relevant studies in the sample may not accurately reflect the total dataset, especially when the sample size is small.

The second stop criterion is based on the number of irrelevant studies that are proposed one after the other. If a certain number of irrelevant studies are suggested in succession without a relevant article being found, the user stops the review. However, for both criteria, the values are unclear. There is no clear answer to the question of how large the sample size should be for calculating the first stop criterion or what percentage of the total dataset should be used as the stop criterion. A third stop criterion, which will also be investigated in the context of this study, is also being discussed on various research platforms and in current research: stopping the screening process after a predefined number of screened studies.

Several studies have already used single stopping rules, either published or presented at conferences. For example, Adamse et al. (2024) [13] used ASReview to screen a dataset of 35,353 studies. They screened a total of 2,908 studies using the ASReview-assisted screening process. The second stop criterion was applied, and it was decided to stop the screening after 100 consecutive irrelevant papers, or about 0.3% of the total dataset. Thus, 32,538 studies were excluded without further screening. As a further quality assurance measure, the reference lists of included studies were screened for additional relevant publications. Notably, Adamse et al. [13] identified additional relevant records among the 32,538 excluded records through this supplementary screening, highlighting the risk of premature stopping. Other studies also used the second stop criterion with values of 5% (corresponding to 40 studies) [14]. For example, other studies use a predetermined value for how much of the dataset is screened (5 or 10–11% per dataset) before screening is stopped [15]. However, it remains unclear why individual values were chosen and whether these values actually represent a reliable criterion for terminating screening. The challenge remains to determine an appropriate criterion for stopping the screening process without risking the exclusion of relevant studies. To date, there is no clear recommendation in this regard (Table 1).

**Table 1:** Possible criteria for stopping the screening process discussed in the literature (1–3)

**Stop Criterion 1**: Stop screening when the calculated number of relevant studies has been reached.	**Stop Criterion 2**: Stop screening when a predefined number of X irrelevant studies are suggested in succession without any further relevant studies being found.	**Stop Criterion 3**: Stop screening when X% of the dataset has been screened.

Note. Bold text indicates the designated label for each stop criterion as referenced throughout the text

This study aims to address this research gap. First, using the retrospective simulation data, it will determine the theoretical upper bound of effort that could be saved in T&A screening by stopping immediately after the last relevant abstract has been identified. This question is of purely theoretical value, as in any prospective systematic review the number and position of relevant studies is unknown; researchers cannot know when the last relevant study has been screened. Nevertheless, establishing this theoretical maximum is methodologically important: it serves as a benchmark against which the practical stop criteria can be compared, allowing us to quantify how close each criterion comes to the ideal stopping point. Therefore, it is necessary to evaluate the above mentioned stopping criteria. The second research question investigates which criteria can be used to stop the T&A screening in ASReview early, without excluding relevant studies. The following hypotheses are tested:

An appropriate stop criterion for a review process in which no relevant studies are erroneously omitted, is….

- Stop criterion 1: A calculated number of relevant studies, based on a dataset sample.
- Stop criterion 2: A specified number of successive irrelevant studies with no relevant studies being erroneously excluded.
- Stop criterion 3: A quantity-based approach in which a predefined percentage of the total dataset is reviewed.

## Methods

**Fig. 1:**
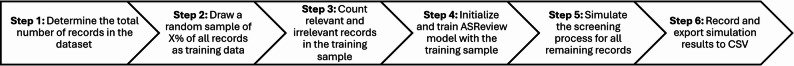
Simulated screening process in ASReview using a Python script

To answer the above questions, a total of 35,000 automated T&A screenings were carried out (7,000 per dataset) on five datasets using ASReview (version 1.6.1) and a Python script written for this purpose (see Supplementary File 2). Each simulation followed the steps illustrated in Fig. 1. To begin, a random subset of studies was drawn from one of the five datasets (see Table 2). This subset represents the initial set of manually reviewed studies that ASReview needs in order to “learn” which study characteristics are associated with relevance – functioning like a training sample for the algorithm. The size of this initial subset was systematically varied across simulation runs, covering seven levels: 1%, 5%, 10%, 15%, 20%, 25%, and 30% of the total dataset. These values were selected to reflect a realistic range of starting conditions, from a minimal initial review effort (1%) to a more extensive one (30%), enabling an assessment of whether the size of the training sample influences the reliability of the stop criteria. Within each subset, studies were classified as relevant or irrelevant. These classifications were then used to train the ASReview model, which subsequently ranked all remaining, unreviewed studies in descending order of predicted relevance. For each combination of dataset and initial subset size, 1,000 independent simulations were conducted. Because the active learning algorithm is re-initialized at the start of each simulation, results varied between runs, reflecting the natural variability that would occur in real-world use. Across all five datasets, this yielded a total of 35,000 simulations (7,000 per dataset).

The simulation data were subsequently used to evaluate the three stop criteria retrospectively – that is, knowing which studies were truly relevant, we could assess whether each criterion would have terminated screening at the right point. For stop criterion 1, the number of relevant studies estimated from the initial training subset was compared to the actual number of relevant studies in the full dataset. The relative difference between these two figures indicates how accurately stop criterion 1 predicts the true number of relevant studies. For stop criterion 2, we identified the largest gap – measured as the number of consecutively screened irrelevant studies – between any two relevant studies in each simulation. This gap represents the minimum threshold at which stop criterion 2 would have operated without missing any relevant study. For stop criterion 3, we recorded the position of the last relevant study within the ranked dataset and expressed this as a percentage of the total dataset size. By examining the variability of this value across datasets and simulations, we assessed whether a single universal percentage threshold could be meaningfully defined.

The SYNERGY dataset [16], a freely accessible and open dataset that shows the study selection in systematic reviews, was used for the analysis. The data set comprises 169,288 academic papers from 26 systematic reviews. In the SYNERGY dataset, the relevance classification of each study, i.e. whether it was included or excluded, was determined by the original authors of the respective systematic review, following their pre-specified inclusion and exclusion criteria. These classifications serve as the ground truth in the present simulation study. Five reviews were selected for this study, each examining different numbers of studies with a different percentage of matching studies. Table 2 shows the names of the studies, the topics, the total number of studies, the number of relevant studies and the percentage of relevant studies. From here on, the reviews will be referred to as the “dataset” since they are considered a dataset with a varying number of different studies and were used as the basis for the simulations. The research questions and PICO/PECO frameworks of the five selected reviews are provided in Supplementary Table S1, as differences in topic complexity and study homogeneity may influence the performance of stopping criteria [17–21].

**Table 2:** Overview of the datasets used for the simulation of the SYNERGY dataset

Name of dataset	Subject area	Number of studies	Number of relevant studies	Percentage of relevant studies
Appenzeller-Herzog (2019) [17]	Medicine	2,873	26	0.9
Bos (2018) [18]	Medicine	4,878	10	0.2
Hall (2012) [19]	Computer Science	8,793	104	1.2
Donners (2022) [20]	Medicine	975	74	7.6
Van der Waal (2022) [21]	Medicine	1,970	33	1.7

## Results

The use of ASReview for T&A screening was evaluated with regard to the proportion of the dataset that needs to be screened to identify all relevant studies. This proportion varied substantially between datasets.

All datasets showed a reduced percentage of records that needed to be screened to identify all relevant studies, compared to manual screening (100%). However, the extent of this reduction varied greatly between datasets. The strongest reduction was observed in the “Bos ( 2018)” [18] dataset, whereas in “Donners (2022)” [20] the reduction was minimal, with up to 94.5% of the dataset still requiring screening. In this specific case, around 95% of the dataset needed to be screened, which wouldn’t have added much value compared to a full screening. Overall, it can be seen that relevant studies are found with less effort on average across all datasets and all simulations than in manual screening processes without ASReview.

To test stop criterion 1, the data on the estimated relevant studies is compared with the actual value. The relative difference between the actual value and the assumed value is shown in Table 3. When considering the different samples for determining the assumed number of relevant studies (Table 3), it can be seen that although the deviations from the actual value do decrease with an increasing number of studies used to train ASReview, they are still present. When considering the use of stop criterion 1 across all categories, it can be seen that stop criterion 1 works reliably in only 2% of the simulations. In about 40% of the simulations, more studies were assumed than were actually available. In 22% of the simulations, 1 study was erroneously excluded and in 36% of the simulations, more than 1 study was excluded. In one simulation, 84 relevant studies were not identified because the stop criterion 1 was used too early.

**Table 3:** Relative distance between true and estimated number of relevant studies per dataset and sample size

Dataset/% Initial screening	1%		5%		10%		15%		20%		25%		30%	
	M	SD	M	SD	M	SD	M	SD	M	SD	M	SD	M	SD
1	76.4	17.6	5.2	17.1	1.9	15.1	0.7	11.6	-0.1	10.2	-0.1	8.8	0.3	7.7
2	90.6	7.7	11.8	6.4	3.3	6.6	1.9	6.4	0.6	5.8	0.4	5.2	0.4	4.5
3	39.5	73.2	0.0	44.7	-0.3	30.6	0.4	23.5	0.6	20.3	-0.2	16.9	1.4	15.7
4	41.2	43.7	0.5	34.1	1.6	25.0	0.8	20.0	1.2	15.8	0.1	14.7	-0.3	12.5
5	69.9	21.1	2.9	20.7	0.1	16.3	0.4	14.0	0.2	11.1	-0.1	9.9	0.4	8.2
Total	66.7	46.3	7.3	29.2	4.5	21.6	4.0	17.4	3.7	14.9	3.2	13.4	3.6	12.3

*M *Mean, *SD *Standard deviation

1 = Appenzeller-Herzog( 2019) [16]; 2 = Bos (2018) [18]; 3 = Hall (2012) [19]; 4 = Donners ( 2022) [20]; 5 = van der Waal (2022) [21]; total = all data sets

To check stop criterion 2, the largest deviations between two matching studies were examined. When examining the largest gaps between two consecutive relevant studies across all simulations, it is apparent that there is no consistent value that can be accepted for stop criterion 2 and that works for all examined datasets and all simulations, irrespective of the training sample size, while at the same time being close to the optimal value for the individual dataset. Supplementary Table S1 presents the results disaggregated by training sample size, which further illustrates the variability in stop criterion 2 performance. For example, the functional stop criterion was identified at 2% in the Bos data set, whereas in the Appenzeller-Herzog data set it was identified at an average of 31%.The standard deviations and maximum values also show a significant difference. If the highest value of over 60% from the Donners dataset were chosen, this would specifically mean that the entire dataset always has to be screened unless all relevant studies are found in less than 40% of the dataset, which, according to Table 4, would only be the case for two out of five datasets and, at the same time, only for one dataset with a relevant screening time saving. Therefore, hypothesis 2 must also be rejected, since no suitable stop criterion 2 can be found that works reliably across different datasets (Table 5).

**Table 4:** Screening percentage to identify all relevant studies per dataset (*n* = 7,000 simulations each)

Review name	*n*	Min	Max	Mean	SD
Appenzeller-Herzog (2019) [17]	7,000	4.2	60.1	43.1	15.0
Bos (2018) [18]	7,000	0.1	4.4	2.9	0.9
Hall (2012) [19]	7,000	1.8	22.4	13.0	5.2
Donners (2022) [20]	7,000	18.9	94.5	76.9	13.3
Van der Waal (2022) [21]	7,000	6.3	53.3	38.2	11.0

*n* denotes the number of simulations that were carried out per dataset

**Table 5:** Values for stop criterion 2 that would work in the respective simulations, in % per dataset

Dataset	Min	Max	M	SD
Appenzeller-Herzog (2019) [17]	0.03	49.0	31.3	12.4
Bos (2018) [18]	0.02	3.0	2.0	0.9
Hall (2012) [19]	0.1	16.0	9.6	4.7
Donners (2022) [20]	0.1	61.0	14.0	17.2
Van der Waal (2022) [21]	0.05	38.0	23.0	9.0

*n* per data set = 7.000

*Min* Minimum value, *Max* Maximum value, *M* Mean value, *SD *Standard deviation; values in percent

Stop criterion 3, which is based on screening a fixed percentage of the dataset, must also be rejected. As already shown in Table 4, the proportion of studies to be screened varies extremely in the different datasets. Therefore, no overall value can be given for stop criterion 3, in which all relevant studies were always included in all datasets.

## Conclusion

This study evaluated three stop criteria for T&A screening in ASReview using retrospective simulations across five datasets. The results show that the screening workload could be significantly reduced across all datasets if a suitable criterion were found to reliably stop the screening. In any case, all relevant studies could be identified earlier compared to a manual screening of the entire dataset. However, the amount of effort saved varies depending on the dataset structure, and for smaller datasets it makes less sense to use ASReview to save effort, since here, as with the “Donners (2022)” [20] dataset, almost 95% of the data set would have to be screened in the least favorable run. Furthermore, no overall value could be found for any of the stop criteria tested that proved useful in all the data records tested. Therefore, the added value in an early identification of all relevant studies can be described as low, since there is no reliable criterion that ensures the screening process can actually be stopped without possibly excluding relevant studies. When examining the values, it could be assumed that in most cases, with a stop criterion 3, all relevant studies were found in the amount of 40 to 45%. However, in the current study, there are enough simulations where this is not the case. It is therefore questionable whether such a stop criterion should be applied. The use of stop criterion 2 in the various studies at 0.03% [13] or 5% [14] seems highly questionable, since a significantly higher value is assumed in the simulations (see Table 5). Based on the results, stop criterion 1 can be rejected. Therefore, it is quite questionable whether the added value of ASReview in terms of reducing the screening workload, as presented in individual studies [9, 10], is achievable if there is no reliable criterion for stopping the screening. These findings are consistent with critiques raised by Callaghan and Müller-Hansen (2020) [11], who demonstrated statistical limitations of fixed stopping criteria, and Callaghan et al. (2024) [22], who argued that universal stopping criteria are unlikely to be sufficient without considering additional parameters such as dataset characteristics and topic complexity. Importantly, the results of this simulation study, which are based on retrospective data with known relevance labels, cannot be directly applied to prospective systematic reviews where the number of relevant studies is unknown. In practice, researchers would need to combine stopping criteria with additional quality assurance measures, such as screening of reference lists or consultation of subject matter experts, to mitigate the risk of missing relevant studies.

However, the use of software for T&A screening can also be regarded as less critical. In the review with ASReview, individual values or processes could be defined for which many, but not all, relevant studies are identified. The question here is more about the costs and benefits of ASReview. If in a large data set of several thousand studies, (almost) all studies can be identified in the first few percent, but one or two studies fail to be identified, it is questionable whether such a small margin of missed studies is acceptable. This consideration is particularly relevant given that even in conventional dual-reviewer screening processes without software, some degree of human screening error is expected when processing thousands of studies, which is precisely why independent screening by two or more reviewers is recommended in established review standards. ASReview may therefore be used in a targeted manner but stop criteria should be applied more critically and the stop criteria used should be set significantly higher than is currently the case.

The present study has some limitations. Firstly, publicly available datasets were used that had already been used in other projects in ASReview. Therefore, the results could be different with new datasets that ASReview does not know. Furthermore, due to the scope of this study, only the default and most established model in ASReview was tested, and not other available calculation models. Further studies should therefore also test the stop criteria with other available models and screening options. Finally, it was not checked whether further studies with given stop criteria would have been found retrospectively by, for example, screening the bibliographies, and therefore a stop criterion would not have led to the exclusion of relevant studies.

Overall, there are currently insufficient evidence-based values that would allow a cutoff with absolute certainty. At the same time, however, other criteria should be tested to allow a stop of the screening process, as this could potentially reduce the time required for the T&A screening phase, though the time needed for setting up and training the ML model should also be considered when evaluating overall efficiency gains.

This study shows that ASReview can accelerate the screening process, but no reliable stop criterion was found to consistently reduce effort across datasets. While relevant studies were identified earlier than with manual screening, the reduction in the number of studies to be screened varies, and for smaller datasets (e.g., fewer than approximately 1,000 studies, as illustrated by the Donners (2022) [20] dataset with 975 studies), the benefit is minimal.

None of the tested stop criteria ensured full identification of relevant studies without risk, questioning the practical value of applying simple universal stopping rules in ASReview-assisted screening. However, for large datasets, it may still serve as a useful tool if stop criteria are applied cautiously. Future research should explore alternative models and additional screening strategies.

## Supplementary Information

Supplementary Material 1.

Supplementary Material 2.

## Data Availability

The datasets used and analysed during the current study are publicly available from the SYNERGY repository (https://dataverse.nl/citation?persistentId=doi:10.34894/HE6NAQ). The Python scripts and simulation framework developed for this study are provided in Supplementary File 2 and are also available from the corresponding author on reasonable request.
